# Dr. Louis Isaac Woolf: At the Forefront of Newborn Screening and the Diet to Treat Phenylketonuria—Biography to Mark His 100th Birthday †

**DOI:** 10.3390/ijns6030061

**Published:** 2020-08-03

**Authors:** José Ramón Alonso-Fernández

**Affiliations:** Neonatal Screening Laboratory, Department of Pediatrics, University Hospital Complex (CHUS–SERGAS) and Universidade de Santiago de Compostela, 15705 Santiago de Compostela, Spain; joseramon.alonso@usc.es

## 1. Introduction

In mid 2019, the author reminded the (International Society for Neonatal Screening) ISNS of the happy occasion of the 100th birthday of one of the living pioneers of neonatal screening, Professor Louis Isaac Woolf. Dr. Woolf turned 100 on April 24, 2019. Then ISNS president Professor Rodney Howell from Miami and Professor Graham Sinclair from Vancouver visited Dr. Woolf to commemorate his work [1]. The ISNS invited the author to write a biography of Dr. Woolf, now aged 101.

## 2. Early Career

Louis I. Woolf was born in London. He studied chemistry at University College London and was awarded a Ph.D. in 1945. It was the end of the Second World War and Woolf worked in the pharmaceutical industry at Allen and Hanburys, a company which prepared predigested foods for babies and the starving in post-war Europe.

In 1947, he was awarded an ICI research fellowship at the Hospital for Sick Children, Great Ormond Street, London, where he worked on inherited metabolic disorders, especially those involving amino acids. This was in the Organic Chemical Research Laboratory in the Institute of Child Health.

During 1949, Woolf investigated the possibility of limiting the postulated harmful effects of phenylalanine or its metabolites in phenylketonuria (PKU). He published his findings and further proposals in 1951 [2]. Woolf showed that the idea that glutamic acid could lower blood phenylalanine concentrations was not correct. He suggested that the intellectual failure in PKU could be due to a phenylacetic acid intoxication and pointed to the reduction of phenylalanine as a possible treatment, *‘If the amount of phenylalanine and its degradation products can be reduced, brain function may be normal, whether this leads to recovery or not, will probably depend on the duration of exposure of the brain to the harmful concentration of the substance’*. The concept for a treatment with a suitable food was raised in the 1930s and reported at the 1939 Annual Meeting of the American Chemical Society by Block and Jervis. Woolf’s concept was based on the work of Schramm et al. [3] who described a form of adsorption chromatography for amino acids with active carbon. Casein hydrolysate could be treated with active carbon, thereby retaining aromatic amino acids. If tyrosine and tryptophan were added to this product, a digestible product low in phenylalanine would be obtained.

However, Woolf could not convince the doctors at Great Ormond Street Hospital to put any of the phenylketonuric patients attending the hospital on this diet.

Meanwhile, Dr. Horst Bickel, at the Children’s Hospital in Birmingham, was consulted on March 13, 1951 by the mother of a girl of Irish descent, Sheila Jones, 17 months old. The ferric chloride test was positive on a urine specimen from April 14, 1951. Using paper chromatography of amino acids, Bickel noted a significant increase in phenylalanine in urine and diagnosed a ‘phenylpyruvic oligophrenia’ syndrome or phenylketonuria.

Dr. Evelyn Hickmans, Clinical Chemist in charge of the biochemistry laboratory at the Children’s Hospital, Birmingham and Dr. John Gerrard, a pediatrician, accompanied Bickel on a visit to Dr. Woolf to learn directly from him the details of his proposed treatment [4]. Bickel then took on the arduous task of preparing this diet to Woolf’s design.

The therapeutic trial with Woolf’s diet began in December 1951 eight months after diagnosis and Sheila at two years and two months old. As now is generally known, within a few months, the clinical improvement was remarkable, the young patient learned to crawl, to stand up, and get on chairs. Her hair darkened and the eczema and the peculiar smell disappeared. When Bickel added phenylalanine to the diet (ethically questionable by current standards), she relapsed and all the symptoms returned (see https://www.youtube.com/watch?v=OqZ7QHO5_hs for historic footage).

As recorded in the journal of the University of British Columbia, UBC Reports, Dr. Woolf commented that, *“It must have taken great courage for the doctor to give her a completely unproven diet. I warned that some of the amino acid would have to be given to her in the form of normal food, otherwise the child wouldn’t be able to grow. In the first few years of this treatment for PKU this advice either wasn’t known or wasn’t followed by some other doctors and the results were disastrous”* [5].

In 1953 and 1954, twenty years after Følling’s first article on PKU [6], Bickel published the results of Sheila’s dietary treatment. Bickel, at some point, admitted that he had thought Woolf’s idea that dietary treatment was possible, was extravagant.

Physicians at Great Ormond Street Hospital now agreed to treat three of their PKU patients with Woolf’s dietary therapy under his direction. Their successful results were reported in a 1955 paper [7]. The authors also discussed the economic benefits of early treatment versus the cost of institutionalization of an untreated individual with learning disabilities, recommending the ferric chloride screening test should be applied to a fresh urine sample of every baby or young child with the slightest suspicion of mental retardation.

By 1956 it was already clear that dietary treatment should be started in the first days of life. The first PKU patients had been treated with a solid diet, as they were already older. During 1956, when still at the Hospital for Sick Children, Woolf faced a dietary dilemma with his first infants, a pair of 17-day-old twins, sisters of a 2-year-old PKU girl with serious impairment. One of the twins was diagnosed with PKU, the other was not. Various formulae were tried to produce a liquid diet suitable for the PKU twin. Eventually, whipped cream as a fat base was added to the charcoal treated casein hydrolysate, thus making a milk that the baby willingly accepted [8].

## 3. Woolf Pioneers a Neonatal Early Detection Program in 1957

**Figure 1:**
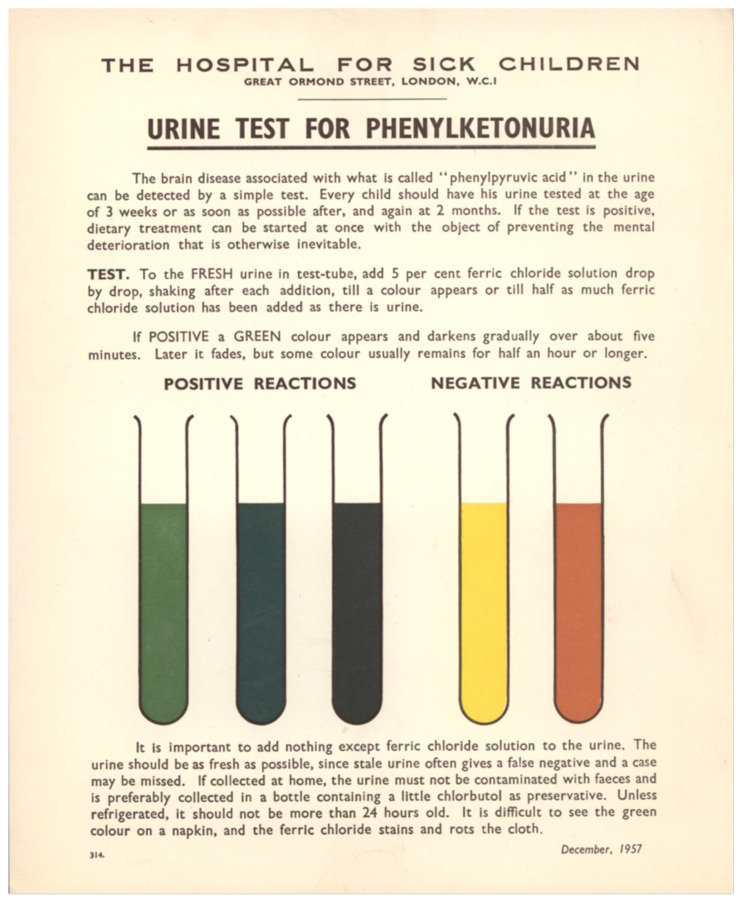
Fact sheet referred to by Woolf et al. in their 1958 article [8]. Thanks are due to the Museum and Archives Service, Great Ormond Street Hospital and its archivist Nicholas Baldwin for providing it and for publishing it in accordance with the Open Government License of the National Archives of the United Kingdom.

In the 1958 paper [8] on the dietary treatment of 11 PKU patients, Woolf et al. pointed out, *“This indicates the necessity for diagnosing phenylketonuria and instituting treatment as early in life as possible. The urine phenylpyruvic assay in urine is extremely simple, and the implications of a positive result are very important”*. To a specimen of fresh urine is added a solution of 5% ferric chloride. A green color that reaches a maximum in a few minutes and fades only slowly was a positive result, an indicative of PKU. Ferric chloride may be dripped on a wet nappy, but the result was less clear then when performed in a test tube (see Figure 1).

In the same paper, the authors for the first time recommended mass screening for this disease, with urine sampled ideally at 21 days after birth. Additionally, they proposed to sample and analyze the urine of siblings of PKU patients at a daily basis.

They recommended that local health authorities made the necessary arrangements for health visitors to collect and test every baby’s urine using the colored card with instructions shown in Figure 1. They also envisioned commencing treatment when a positive screening result was confirmed, an early record of an important screening principle.

## 4. Woolf and the Global Development of PKU Screening

In 1957, W.R. Centerwall [9], in a letter to *JAMA*, reported on the project of the College of Medical Evangelist of Los Angeles, intended to detect cases of PKU in early childhood by adding a drop of ferric chloride-solution to a wet nappy, claiming the idea of a screening program for PKU. In a second letter [10], Centerwall mentioned that Dr. Armstrong, University of Utah, USA, was aware of Centerwall’s project, informed him of a similar program in England. Centerwall described the screening program initiated by Woolf in 1957, and intended to include all English babies. He mentioned the existence of an illustrated booklet describing the assay and its purpose and that it was available to all doctors and clinics in England to be ordered from the Great Ormond Street Hospital. This is brought forward by a few months with the existence of this colorful leaflet to the readers of *JAMA* which Woolf et al. would later report on in the United Kingdom.

In 1958, Baird et al., Philadelphia, USA wrote [11] that the incidence of phenylketonuria is so low at 1 in 20,000 live births that a fast and safe screening test is essential if the diagnosis is to be made before the development of obvious mental retardation. Baird prepared strips of paper impregnated with ferric chloride and glacial acetic acid and allowed them to dry. A positive reaction occurred if the treated portion turned green, grey, or dark blue within 60 s of immersion in the urine to be tested. A footnote indicated that these improved strips were supplied by Ames Laboratories. Ames had been preparing solid phase reagents since 1945. In 1958, Ames launched the Phenistix for PKU screening.

In 1959, N. K. Gibbs and Woolf [12], the latter now working in Oxford, reported on the detection program carried out in Cardiff, UK from 1 March 1958 to 1 March 1959. It could be considered the first official program as it involved the health authority. From December 1958, the infant urines were also tested with Phenistix in addition to the ferric chloride test. Phenistix is a paper strip reagent test used on a child’s urine collected in a bottle with a little chlorbutol as a preservative, at the age of three weeks or as soon as possible, and brought by the mother at a consultation visit. In 1959, 4530 children were born in Cardiff and 1141 analyzed, finding one case of PKU and seven false positives. The false positives were attributed to the interference of p-hydroxyphenylpyruvic acid, and did not occur when the Phenistix was used instead of ferric chloride. Gibbs and Woolf concluded that the greatest difficulty was obtaining the urine and that the Phenistix was superior to using ferric chloride.

In 1958, Helen K. Berry, Cincinnati, USA devised a simple method for collecting urine on paper. As collecting urine specimens from children is always difficult, she and co-workers showed that urine containing phenylpyruvic acid, dried on an adsorbent paper, gave a positive test with the ferric chloride reagent, even after several months [13].

Whatever the method of choice, in the early days of mass testing, several practical issues needed to be resolved.

The local health authority in Birmingham, UK started a PKU detection program in 1959 using Phenistix. [14] The health visitor placed the Phenistix between the folded nappy and squeezed with a wooden spatula for 3 s. The choice of a nappy-test was made because the experience in Cardiff was that it was difficult to collect liquid urine and because the assay with ferric chloride in solution, dripped on the nappy, left a stain that was hard to remove. Attempts to drain urine from the nappy or to analyze filter paper by pressing on the nappy were not satisfactory. In 1959, 19,353 children were born in Birmingham of which 98.8% were screened at six weeks of age. One case of phenylketonuria was found, meeting the expected birth prevalence of 1 per 20,000.

In Edinburgh, UK where there were approximately 8500 newborns per year, the health authority commenced an early detection program for PKU in 1960 [15]. Using Phenistix in nappies, they analyzed 98.2% of children who had reached 6 weeks of age. A first false negative result led the program to reconsider both the sampling and the logistics of the test. Firstly, the sampling was adapted to placing a tissue with a plastic sheet on the genitals of the newborn before putting on the napkin. The tissue, once soaked and following the instructions given to the mothers, was to be stored in a plastic bag to give to the screener to be taken to a centralized laboratory for analysis, preferably within a few hours. Secondly, the logistics were the subject of reconsideration. It was concluded that it was vital to the performance of the test that the analysis should be centralized in a laboratory with experienced personnel who understood the crucial steps in the analytical procedure.

In 1961 Woolf reviewed the existing tests for the detection of phenylketonuria [16], including Phenistix, ferric chloride and 2,4-dinitrophenylhydrazine (which requires liquid urine). He commented on the use of paper impregnated with urine and left to dry, emphasizing that with this specimen (the so-called specimen of Berry–Woolf), assays for various pathologies can be carried out. Woolf did not expect this urine impregnated paper specimen to be used in mass screening in the UK because there were no laboratories in a position to receive specimens from 600,000 newborns per year. He commented that the Berry–Woolf specimen was a convenient and easily transportable sample for confirmatory assays. In Cincinnati, the PKU screening program had already expanded to include proteinuria and galactosuria [17]. Woolf extended it further, adding glycosuria and cystinuria/homocystinuria.

In a letter in 1967 [18], Woolf elaborated on the same points stating:

“In 1963 I began to come across tragic cases of phenylketonuria undetected by Phenistix testing in the neonatal period. It seemed that ‘field’ tests such as Phenistix would have to be replaced by methods where a specimen of blood or urine is sent to a central laboratory for testing by more sophisticated techniques.[..]Eight different laboratory techniques are currently in use: the Guthrie method using blood; urine chromatography for o-hydroxyphenylacetic acid; spectrofotofluorometry of blood; the Guthrie method using urine; and chromatography of amino acids in blood by the methods of Berry, Efron, Scriver or Mellon. All these methods except spectrofotofluorometry, have the great advantage that they test for other inborn errors of metabolism as well as phenylketonuria. Two methods, the Guthrie method using blood and chromatography of urine, have now been used long enough and on a large enough scale for us to say that they meet the criteria for a suitable test. Thanks to the co-operation of the medical officers of health of Buckinghamshire, Cardiff, Oxford, and Oxfordshire, about 25,000 newborn infants have had their urine examined here using chromatography. The advantage of this method is that the mother herself can collect a specimen of her baby’s urine on filter-paper and post it.”

At a Washington Conference on Phenylketonuria in 1966, Woolf [19] presented data on the neonatal screening programs in Britain which were starting, reporting that in May 1962, of the 145 local health authorities in England and Wales, 131 were operating a mass screening program for phenylketonuria and five were actively planning such a program. By June 1963, the urine of 2,400,000 newborns had been tested using Phenistix and 104 cases of phenylketonuria had been discovered, early enough to maximize the effectiveness of the treatment.

## 5. Woolf’s Interactions with Spanish Investigators

In 1966–1967, while Woolf was in Oxford, he was visited by F. Mayor-Zaragoza, Professor of Biochemistry in Granada, who was on an Oxford sabbatical and was staying with Prof. Dr. Hans Krebs. Prof. Mayor-Zaragoza was enthusiastic about Woolf’s ideas on screening and as soon as he had returned in Spain, tried with some difficulty to convince the then Director General of Health. He ultimately succeeded, stressing the importance of a screening program to alleviate the suffering of each individual patient. Eventually, a pilot program in Granada was started. Mayor-Zaragoza sent a film crew from Granada to record Woolf’s techniques, and invited him to give a lecture in Granada which Mayor-Zaragoza translated into Spanish, sentence by sentence. The methodology of Woolf (and Berry) was especially well received in Spain. These assays were employed in 1978 in Santiago de Compostela (Galicia) when introducing neonatal screening. For various reasons, Woolf’s original methodology was adapted (see e.g., reference [20,21]). Other assays (e.g., a cystine/homocystine test or Brand’s test) were performed in the same way as Woolf suggested.

Woolf was supportive of the Spanish initiative to introduce neonatal screening and wrote in one letter to Mayor-Zaragoza, *“I agree with you that it would be very appropriate to develop a neonatal screening service in Spain, particularly if this screening is not confined to phenylketonuria and is also open to other congenital errors of metabolism”*.

In Galicia and also in Murcia, the Berry–Woolf specimen is used to this day determining 124 analytes by tandem mass spectrometry.

## 6. Woolf in Vancouver

**Figure 2:**
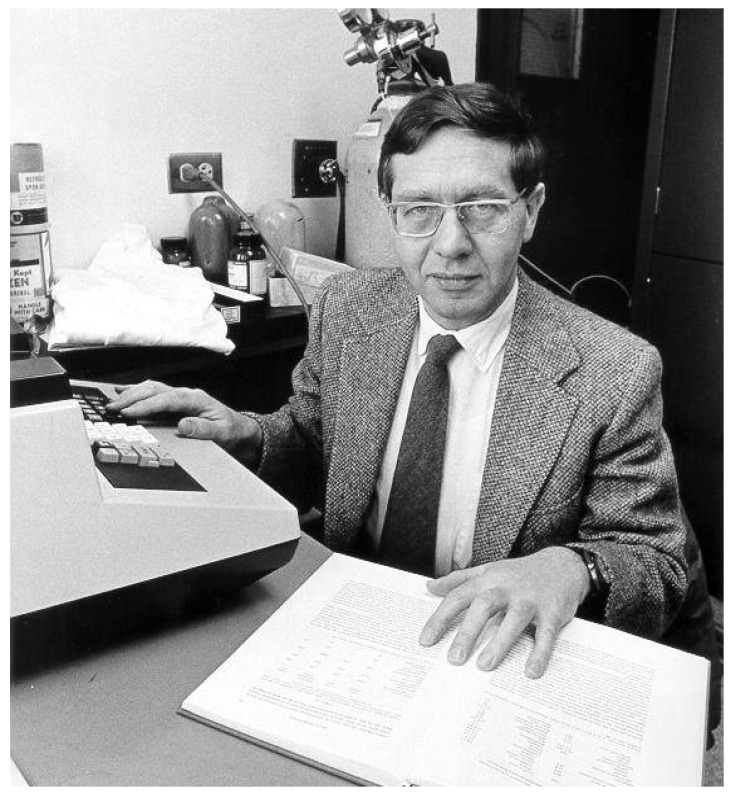
Dr. Louis Woolf in his office in Vancouver 1972 (from University of British Columbia Archives, Photographer Unknown [UBC 41.1/2188]).

In 1968, Woolf joined a group working on neurochemistry and neurophysiology in the Division of Neurological Sciences at the University of British Columbia, Vancouver, BC, Canada. In 1984, he became Professor Emeritus (Figure 2).

In 1968, after he had commenced work in Vancouver, Woolf’s review written in Oxford on Mass Screening of the Newborn for Metabolic Disease was published [22]. Its title indicates that it does not refer exclusively to PKU. The review describes the use of urine samples on paper but also recognizes the value of sampling of newborn blood obtained by heel puncture collected on filter paper, the Guthrie card. Woolf commented that it was universally accepted that analyses should be performed in centralized laboratories and only in that way could operating costs be reduced and experience gained to make the analyses more reliable. The provision of a laboratory covering 50,000 to 100,000 births per year seemed close to the ideal, although geographical factors would also come into play. He added that PKU was only one of a number of inborn errors of metabolism treatable by diet in early infancy and it was possible to include tests for other conditions such as galactosaemia and homocystinuria by chromatography of dried urine and maple syrup urine disease and hyperprolinaemia by chromatography of dried blood.

Woolf began work in Vancouver examining and improving the research methodology of phenylalanine biochemistry in normal and pathological situations, beginning with a careful re-evaluation of an animal model, showing that a mouse, suggested to be a model for PKU, actually was not. Woolf had previously been unable to corroborate serum phenylalanine elevation and now additionally reported failure to detect any of the alterations supposedly attributed to this d1/d1 mouse model of PKU and other hyperphenylalaninemias, attributing these authors’ observations to malnutrition derived from neurological alterations.

In 1971, Woolf edited with H. Bickel and F. P. Hudson the book ‘Phenylketonuria and some other inborn errors of amino acid metabolism’. It was based on the Heidelberg symposium of June 1969. Woolf wrote part of chapter V on the genetics of phenylalaninaemia. In the section Population Genetics, he indicated that a regional high prevalence of PKU can only be explained by a balanced polymorphism, where being heterozygous has some genetic advantage, in the geographical or ethnic environment in which such a high frequency occurs. Woolf continued to have an interest in the cause of this genetic advantage of the PKU-heterozygote, given e.g., a publication in 1986, suggesting that genetically determined higher phenylalanine levels may be selectively protective against certain mycotoxins.

In 1974, Woolf published with colleagues Woo and Gillan work on the isolation of phenylalanine hydroxylase from human [23].and rat liver [24] and its properties.

In 1976, in a review of the congenital metabolopathies that are treated with a diet or a vitamin supplement [25], Woolf covered a wide range of inborn errors of metabolism. These included galactosaemia, phenylketonuria, leucinosis (maple syrup urine disease), valinaemia, isovaleric acidaemia, β-methyl-crotonylglycinuria, propionic acidaemia, hyperprolinaemia, citrullinaemia, d- and l-methylmalonic acidaemias, non-ketotic hyperglycinaemia, arginosuccinic aciduria, types I and II hyperammonaemia, hyperargininaemia, homocystinuria, histidinaemia, cystinosis, hyperlysinaemia, saccharopinuria, and ornithinaemia. Concerning therapy, he covered the use of vitamins at pharmacological doses, for example pyridoxine in glutamate decarboxylase deficiency, in xanthurenic aciduria, about 50% of cases of homocystinuria, and biotin in tiglylglycinuria and in a form of propionic acidemia. Additionally, he discussed defects of vitamin B12 metabolism and transport and errors of folic acid metabolism which respond to vitamin supplementation. Family, social, and psychological problems of special diets are mentioned.

## 7. Woolf’s More Recent PKU Work

In 1979, Woolf published an editorial on phenylketonuria and its variants [26] commenting on the experiences of stopping the diet after a few years, mentioning the possibility that damage to a mature brain produces a spectrum of signs and symptoms different to that of the newborn and that it is necessary to look for indicators sensitive to late intoxication by phenylalanine. He refers to cases of atypical PKU with blood concentrations of phenylalanine below the typical patients with PKU, although considerably higher than normal, the so-called hyperphenylalaninemia variants. In these hyperphenylalaninemias, the phenylalanine hydroxylase activity in the liver is appreciable, although less than in normal. He comments that the range of laboratory findings vary from almost normal to typical of PKU without discontinuities.

Additionally, in 1979, in a letter, Woolf discussed the consequences of interrupting a low phenylalanine diet between five and ten years of age. He indicated that previously well-adjusted patients can be melancholic, dismissive, distracted, and disinterested in their work. Changes in their behavior tend to make them less socially acceptable; they may become irritable, etc., IQ-tests (which were the norm but could produce a false sense of security) may not be the ideal test to evaluate the status of these patients and a series of psychological and psychiatric studies that could be done was presented.

## 8. Epilogue

Dr. Woolf was at the forefront of and an intrinsic and important part of neonatal screening. He led the way to the preparation of the therapeutic diet for phenylketonurics and initiated and justified newborn screening programs, opening a new path in public health and preventive medicine. This biographical paper attempts, not only to address Dr. Woolf’s vital role in the development of a therapy for PKU, but also his work concerning inborn errors of metabolism, diagnosis, and treatment. Many of the screening principles published by Wilson and Jungner in 1968 [27] were recognized in the work and ideas of Woolf. This paper also tries to acknowledge just that. This paper deals with only part of Dr. Woolf’s work; for further reading see [20,21].
